# Case Report: Post-Partum Complications of *NFκB1* Deficiency Underscore a Need to Better Understand Primary Immunodeficiency Management During Pregnancy

**DOI:** 10.3389/fped.2021.648022

**Published:** 2021-07-07

**Authors:** Diem-Tran I. Nguyen, Amanda Grimes, Donald Mahoney, Sebastian Faro, William T. Shearer, Aaron L. Miller, Nicholas L. Rider

**Affiliations:** ^1^Department of Pediatrics, Baylor College of Medicine, Houston, TX, United States; ^2^Section of Hematology and Oncology, Baylor College of Medicine and Texas Children's Hospital, Houston, TX, United States; ^3^Department of Obstetrics and Gynecology, Women's Hospital of Texas, Houston, TX, United States; ^4^Section of Immunology, Allergy and Retrovirology, William T. Shearer Center for Human Immunobiology, Texas Children's Hospital, Baylor College of Medicine, Houston, TX, United States; ^5^Department of Pediatrics, University of Texas Medical Branch, Galveston, TX, United States

**Keywords:** NFκB1, inborn errors of immunity, primary immunodeficiencies, pregnancy, pregnancy complications

## Abstract

Nuclear factor κappa-B (NFκB) is a family of transcription factors involved in regulating inflammation and immunity. Mutations in the *NFκB1* pathway are associated with primary immune defects and underlie the most common monogenic etiology of common variable immunodeficiency (CVID). However, little is known about how *NFκB1* defects or primary immunodeficiency (PID) complicate pregnancy. We present a previously healthy 34-year-old patient who suffered from poor wound healing and sterile sepsis during the post-partum period of each of her three pregnancies. She was otherwise asymptomatic, but her daughter developed Evans Syndrome (ES) with hypogammaglobulinemia prompting expanded genetic testing which revealed a novel monoallelic variant in *NFκB1*. This case highlights that pregnancy-related complications of PID can be difficult to recognize and may portend adverse patient outcomes. For these reasons, guidance regarding diagnosis and management of women of childbearing age with PID is warranted.

## Introduction

Nuclear factor κ-B (NFκB) is a family of transcription factors involved in the regulation of numerous cellular pathways that mediate inflammation (1) and immunity (2). The NFκB pathway plays an important role in both adaptive and innate immunity including hematopoiesis, lymphoid organogenesis, and cellular signaling in response to pathogen recognition (2). Derangement of the NFκB pathway has been associated with several immunodeficiences (3–9). Specifically, truncating, missense, and deletions within *NFκB1* have been implicated as the most common monogenic etiology of common variable immunodeficiency (CVID), accounting for 4% (16/390) cases in a large European cohort (10). These mutations result in heterozygous loss-of-function variants leading to *NFκB1* haploinsufficiency. Among patients with *NFκB1* haploinsufficiency there is a wide phenotypic spectrum with variable expressivity even within the same family unit (10). Clinical presentations may include recurrent infections, autoimmune disease, and malignancy; in particular, Evans Syndrome (ES) should trigger concern for *NFkB1-*related disease (11–13). Notably, the phenotypic features continue to evolve as a description of recurrent necrotizing cellulitis following a dental procedure has just been described within the spectrum of *NFkB1* deficiency (14).

Despite tremendous advancements in understanding the biology and management of primary immunodeficiency (PID) relatively few reports focus on how PID manifests in or complicates pregnancy. In a study surveying 590 women with primary humoral immune deficiency, only 15% were diagnosed prior to their first pregnancy (15). This study suggests that the majority of women lack a diagnosis of PID prior to pregnancy and thereby may have hidden health risks unbeknownst to them and their healthcare providers. This could lead to suboptimal outcomes. For example, a Czech study found that patients with CVID were more likely to suffer from vaginal bleeding, pre-eclampsia, eclampsia, preterm labor, and were more likely to deliver low birth weight babies than the general population (16). Another study found that patients with CVID have increased risk of fetal loss (15). Additionally, pregnant patients with PID may warrant altered treatment regimens. In a study examining CVID, IgG replacement therapy was often increased during pregnancy to not only protect a mother but also her fetus with transplacental IgG (15).

Here, we report a patient with *NFκB1* deficiency who suffered sepsis and impaired wound healing during each of her three pregnancies. Our patient's first daughter developed ES and was diagnosed with *NFκB1* deficiency leading to the mother's diagnosis. To the best of our knowledge, there are no documented cases of *NFκB1* deficiency presenting during pregnancy. This case of familial *NFκB1* deficiency underscores risks associated with PID and adds to the literature regarding unique concerns related to pregnancy among PID patients.

## Materials and Methods

### Patient Demographic and Clinical Information

We conducted a retrospective chart review of electronic medical records from outside facilities and Texas Children's Hospital in Houston. Our study was performed in accordance with a Baylor College of Medicine IRB-approved protocol (H-21453) and with the patient's consent.

### Testing

Testing on the patient and her daughter included comprehensive lymphocyte phenotyping, proliferation studies, quantitative immunoglobulin measurement, and routine labs. Trio whole-exome sequencing (WES) was performed commercially by GeneDx, Inc. (Gaithersburg, MD) on the patient and patient's daughter.

### Clinical and Molecular Findings

**Figure 1:**
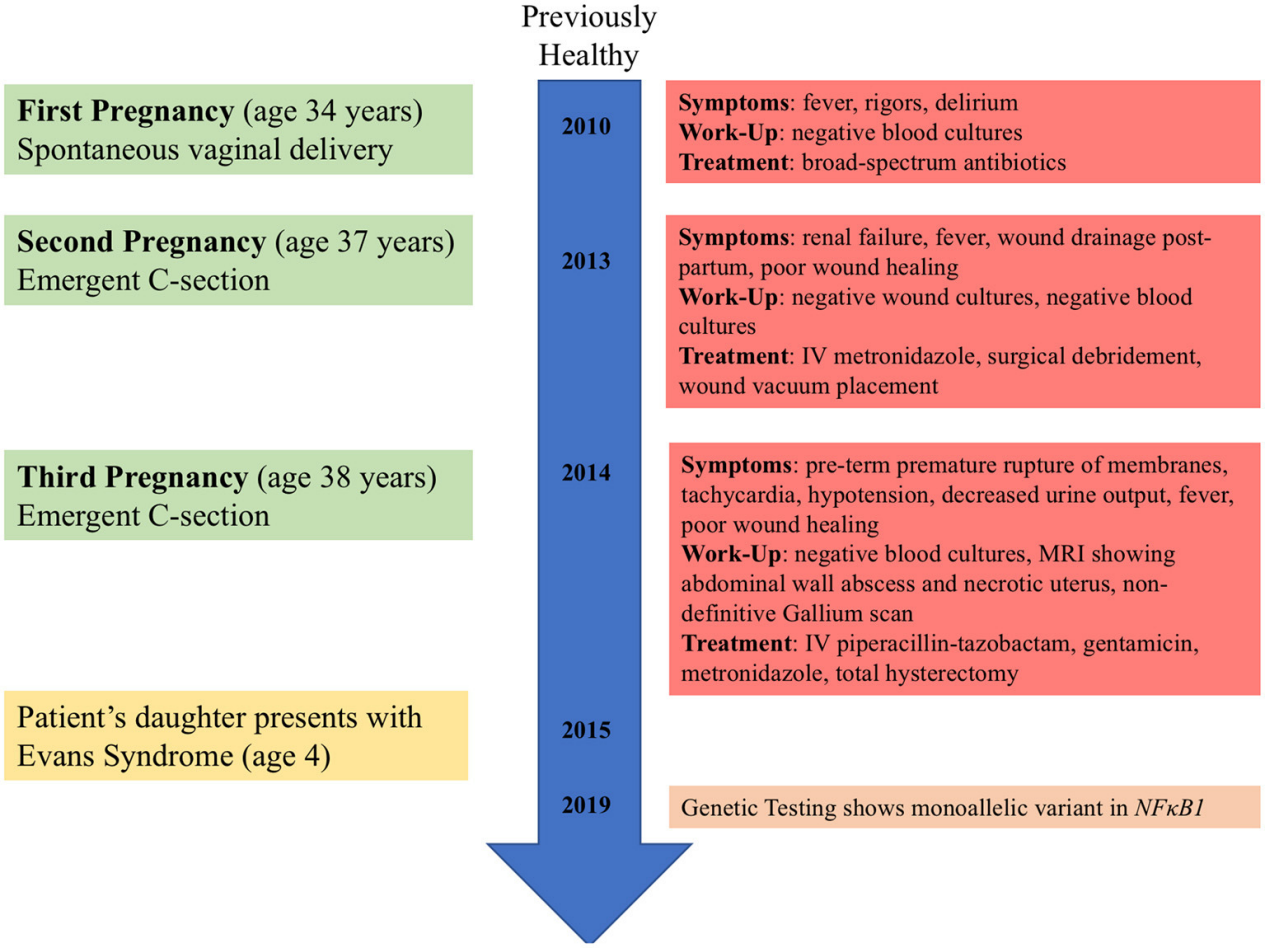
Timeline of clinical events for patient and patient's daughter.

**Figure 2:**
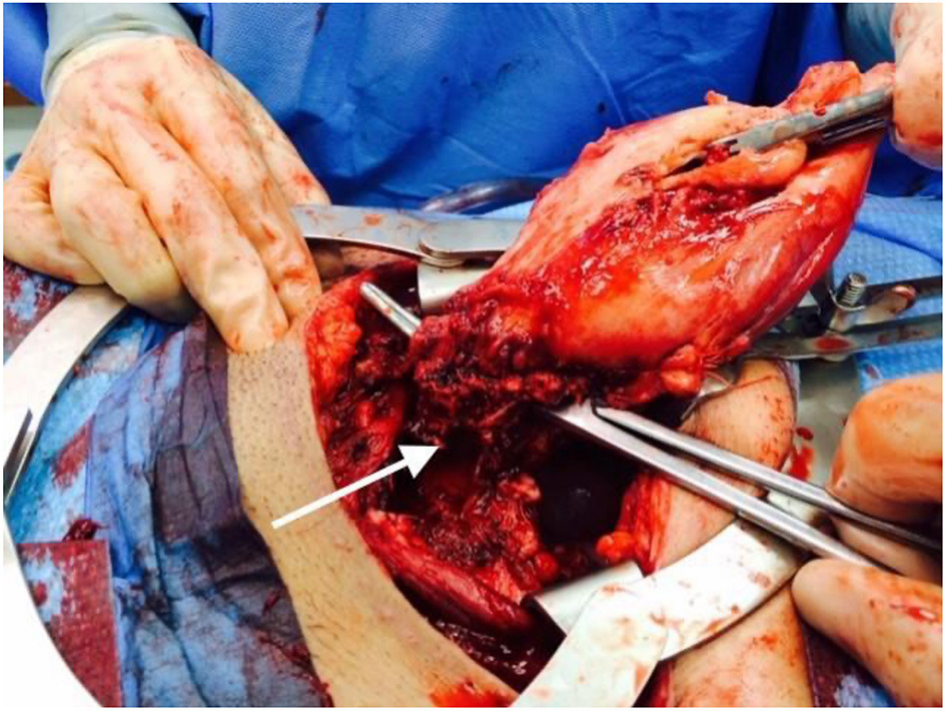
Intraoperative photo from 2014 total hysterectomy. The arrow points to a necrotic area at the uterine base.

The patient's high-level clinical course is shown in Figure 1. She is an otherwise healthy female, without significant prior infectious or inflammatory complications, who presented in her thirties with three post-partum episodes of sterile sepsis. She has not suffered infertility concerns or any pregnancy losses. Her first pregnancy resulted in spontaneous vaginal delivery but was complicated by fevers, rigors, and delirium, which seemed to improve with empiric IV antibiotics despite a non-revealing infectious work-up. Her second pregnancy required emergent C-section for fetal distress due to nuchal cord and was complicated by culture-negative decompensated sepsis and acute renal failure which resolved fully. Furthermore, she had poor wound healing requiring surgical debridement and vacuum-assisted closure. Her third pregnancy again required emergent C-section for pre-term premature rupture of membranes with subsequent sterile sepsis. Pelvic imaging suggested an abdominal wall abscess and exploratory laparotomy revealed necrosis of the uterus (Figure 2), requiring total hysterectomy. The patient has since fully recovered without subsequent medical concerns.

**Figure 3:**
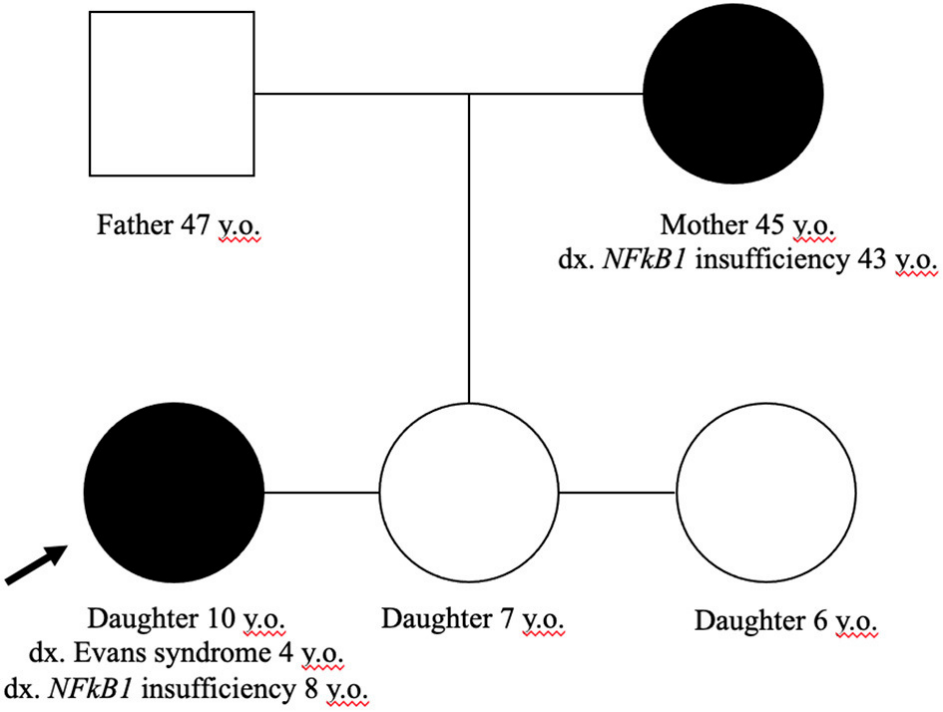
Family pedigree. Only the mother and affected daughter harbor the *NFkB1* (IVS2-2A>T; c.40-2A>T) variant. The father and other siblings have no evidence of altered immunity.

Our adult female patient was diagnosed with *NFκB1* deficiency via expanded familial genetic testing (Trio WES) after molecular confirmation of her daughter's disease. Her extended family history was not suggestive of any parent or siblings with disease; however, her parents and siblings have thus far declined confirmatory testing. Further details about the patient's daughter and her initial clinical presentation prior to understanding the molecular cause was previously reported (17). The noted familial variant in our family is *NFkB1* (IVS2-2A>T; c.40-2A>T) which has not been previously reported, and was described as a variant of uncertain significance (VUS). The change is predicted to impair normal splicing of Exon 2. Given the daughter's presentation of ES and hypogammaglobulinemia in association with the *NFkB1* variant without other notable findings on WES, plus appropriate familial segregation, we believe this variant has sufficient biological plausibility. Her unaffected father and siblings did not harbor the variant (Figure 3).

In addition to the dissimilar clinical features between mother and daughter in our family, laboratory findings were also notably different (Table 1 and Supplementary Table 1, patient's daughter from diagnosis at 4 years through 9 years) in that hypogammaglobinemia and cytopenias were noted in the daughter; whereas, the mother's laboratory features were largely normal (Table 1) with the exception of subnormal pneumococcal titers to 14 serotypes (data not shown). The mother does not have a known history of cytopenias; whereas, her daughter presented with ES. For example, the daughter's blood count at 4 years of age was notable for leukopenia and thrombocytopenia. Additionally, the mother has never displayed immunoglobulin deficiency or signs of lung disease on CT; while, the daughter requires IgG replacement therapy for hypogammaglobulinemia.

**Table 1:** Relevant immunologic findings for patient.

**Laboratory parameter**	**Reference values**	**Patient (age 43 years)**
WBC (×10^∧^3/μl)	4.5–11	8.22
RBC (×10^∧^6/μl)	4.2–5.4	5.08
Hgb (g/dl)	12–16	14.9
HCT (%)	36–46	42.9
Platelet (×10^∧^3/μl)	150–450	195
ANC (×10^∧^3/μl)	1.8–7.7	4.84
IgA (MG/DL)	66–295	52.7
IgG (MG/DL)	641–1,353	775
IgM (MG/DL)	40–80	72.4
Absolute lymph count (10^∧^3/μl)	10^∧^3/μl	2,706
CD3+T cell percent (%)	62–89	74.5
CD3+T cell number (10^∧^3/μl)	551–2,500	2,017
CD3+CD4+percent (%)	32–70	39.8
CD3+CD4+number (10^∧^3/μl)	246–1,811	1,077
CD3+CD8+percent (%)	7–35	28.3
CD3+CD8+number (10^∧^3/μl)	65–850	766
CD19+B cell percent (%)	6–19	14.4
CD19+B cell number (10^∧^3/μl)	38–487	390
CD3-CD56CD 16+percent (%)	2–23	10.8
CD3-CD56CD 16+number (10^∧^3/μl)	45–406	292
Proliferation to mitogens and antigens	NA	Normal Relative to Control and Lab Standard

## Discussion

In this study, we present a patient with *NFκB1* haploinsufficiency whose disease was unmasked by pregnancy, resulting in poor wound healing and sterile sepsis. Despite the severity of these complications, a clear immune defect was not discernable. However, the predicted pathologic variant with clear penetrance in the patient's child coupled with a well-described clinical heterogeneity of disease strongly argue in favor of *NFkB1*-related disease in our adult patient. This case highlights that complications of PID in pregnancy can be under-recognized and that unusual infectious or inflammatory complications should prompt consideration of PID within any clinical context. We expect this description to enhance awareness about PID for healthcare providers in Obstetrics, Gynecology as well as any generalist or specialist who cares for women of child-bearing age. Furthermore, there remains little understanding of the relationship between pregnancy and immune homeostasis in patients with immunodeficiency.

While all forms of PID may impact pregnancy, *NFkB1*-related disease may have specific consequences. The NFκB pathway has been reported to play different roles in pregnancy in the stages of implantation, maintenance, and labor (18). During implantation, NFκB is upregulated to expand the expression of inflammatory factors secreted by natural killer cells, decidual cells, and lymphocytes in the endometrium. These changes act to degrade the extracellular matrix and enable trophoblast invasion. The maintenance phase is characterized by the depletion of NFκB, which in turn results in reduced T-cell production of cytokines. This reduction in cytokines facilitates the immunosuppression required for maternal-fetal tolerance. In labor, increased NFκB influences the regulation of cytokines in the uterus, amniotic fluid, and placenta which result in inflammation, fetal membrane remodeling and cervical ripening, and uterine contraction. Our patient's monoallelic variant appears to have had the largest impact during the labor phase of her pregnancy. It is unclear if physiological processes of the other pregnancy stages are less affected by loss-of-function *NFκB1* mutations or that specific mutations may dictate the timing and type of complications. In mouse models, inactivation of *NFκB1* does not appear to impact fertility as knock-out mice were capable of reproducing normally (19, 20).

In addition, *NFκB1* is implicated as a messenger of keratinocyte proliferation. One study examined the interplay between histone demethylase JMJD3 and NFκB in wound healing, and found impaired keratinocyte migration in NFκB-inactivated keratinocytes (21). This role of NFκB may explain why this patient presented with poor wound healing after multiple labor-intensive pregnancies, and had extended recovery time post-surgery with her Cesarean deliveries. Lastly, the recent report of recurrent necrotizing cellulitis in a patient with *NFkB1* deficiency supports the notion that our patient and that patient reveal a previously under-reported feature of the phenotypic spectrum of this disease (14).

## Conclusions

Pregnancy-related complications of PID are underreported and underrecognized. Given the physiological demands of pregnancy, delivery and recovery, practitioners should maintain vigilance for inborn errors of immunity when women suffer unusual complications during this vulnerable time. Undiagnosed PID could portend adverse outcomes for women during pregnancy and delivery. Patients with known PID should receive coordinated care by an expert clinical immunologist and her obstetrician throughout gestation and following delivery. We believe this case of *NFκB1* deficiency may offer insight into the presentation, complications and management of PID during pregnancy. Future considerations of guidance for women of childbearing age with PID are warranted in a systematic fashion.

## Data Availability Statement

The original contributions presented in the study are included in the article/Supplementary Material, further inquiries can be directed to the corresponding author/s.

## Ethics Statement

The studies involving human participants were reviewed and approved by Baylor College of Medicine. Written informed consent to participate in this study was provided by the participants' legal guardian/next of kin. Written informed consent was obtained from the individual(s), and minor(s)' legal guardian/next of kin, for the publication of any potentially identifiable images or data included in this article.

## Author Contributions

D-TN, AM, AG, DM, SF, and NR obtained and analyzed the clinical data. D-TN, AM, and NR wrote the manuscript. AM, AG, and DM aided with manuscript edits. SF contributed figures. WS provided initial care and evaluation of the family presented. All authors contributed to the article and approved the submitted version.

## Conflict of Interest

The authors declare that the research was conducted in the absence of any commercial or financial relationships that could be construed as a potential conflict of interest.
