# Supplementary material for: Case Report: Post-Partum Complications of NFκB1 Deficiency Underscore a Need to Better Understand Primary Immunodeficiency Management During Pregnancy
Source: Front Pediatr. 2021 Jul 7;9:648022. doi: 10.3389/fped.2021.648022 (PMC8292645; doi:10.3389/fped.2021.648022)
Supplement: Supplementary file 1 [file Table_1.docx]

Supplemental Table 1:. Relevant Immunologic Findings for Patient’s Daughter

| Laboratory Parameter | Reference Values | 4 years | 5 years | 6 years | 7 years | 8 years | 9 years |
| --- | --- | --- | --- | --- | --- | --- | --- |
| WBC (x 10^3/µL) | 4-5 y.o 5.0-14.5  6-7 y.o. 5.0-14.5  8-9 y.o. 5.0-14.5 | 2.6 | 5.78 | 4.53 | 3.79 | 4.8 | 5.11 |
| RBC (x 10^6/µL) | 4-5 y.o. 3.9-5.3  6-7 y.o. 3.9-5.3  8-9 y.o. 4.0-5.2 | 4.43 | 5.17 | 5.15 | 5.01 | 5.46 | 5.38 |
| Hgb (g/dL) | 4-5 y.o. 11.5-14.5  6-7 y.o. 11.5-14.5  8-9 y.o. 11.5-15.5 | 12.2 | 14.3 | 14.2 | 13.4 | 14.7 | 13.9 |
| HCT (%) | 4-5 y.o 34.0-40.0  6-7 y.o. 34.0-40.0  8-9 y.o. 35.0-45.0 | 33.8 | 41.1 | 40.1 | 39.4 | 43.2 | 41.4 |
| Platelet (x 10^3/µL) | 4-5 y.o. 135-361  6-7 y.o. 150-450  8-9 y.o 150-450 | 10 | 82 | 122 | 168 | 195 | 196 |
| ANC (x 10^3/µL) | 4-5 y.o 1.9-10.3  6-7 y.o. 1.5-8.0  8-9 y.o. 1.5-8.0 | 0.12 | 1.51+ | 1.61 | 1.01 | 1.12 | 1.68 |
| IgA (MG/DL) | 4-5 y.o. 25-154  6-7 y.o. 32-191  8-9 y.o. 42-223 | 13 | TNP | 9.5 | 7.4 | 7.6 | <6.0 |
| IgG (MG/DL) | 4-5 y.o 463-1,236  6-7 y.o. 635-1,284  8-9 y.o. 610-1,577 | 366 | 498* | 511* | 421* | 1,110* | 1,010* |
| IgM (MG/DL) | 4-5 y.o. 43-196  6-7 y.o. 44-190  8-9 y.o. 48-222 | 18 | TNP | 11.1 | 10.3 | 13.4 | 19.9 |
| Absolute Lymph Count (10^3/µL) | 10^3/µL | 2,880 | 2,784 | 2,360 | 1,672 | 1,968 | 1,887 |
| CD3+T Cell Percent  (%) | 4-5 y.o. 58-74  6-7 y.o. 56-76  8-9 y.o. 56-75 | 68 | 70 | 73 | 72.4 | 72.6 | 71.3 |
| CD3+T Cell Number (10^3/µL) | 4-5 y.o. 1,656-3,841  6-7 y.o. 991-2,997  8-9 y.o. 1,200-2,600 | 1,958 | 1,949 | 1,723 | 1,211 | 1,429 | 1,345 |
| CD3+CD4+Percent  (%) | 4-5 y.o. 28-47  6-7 y.o. 25-48  8-9 y.o. 31-47 | 35.6 | 40.3 | 42.3 | 42.6 | 41.4 | 38.7 |
| CD3+CD4+Number (10^3/µL) | 4-5 y.o. 871-2,379  6-7 y.o. 635-1,620  8-9 y.o. 650-1,500 | 1,025 | 1,122 | 998 | 712 | 815 | 730 |
| CD3+CD8+Percent (%) | 4-5 y.o. 16-32  6-7 y.o. 16-43  8-9 y.o. 18-35 | 25.9 | 22.9 | 22.9 | 21.6 | 22.7 | 25.5 |
| CD3+CD8+Number (10^3/µL) | 4-5 y.o 518-1,433  6-7 y.o. 293-1,221  8-9 y.o. 370-1,100 | 746 | 638 | 540 | 361 | 447 | 481 |
| CD3+/CD4-/CD8-Percent (%) |  |  |  |  | 7.0 | 7.9 | 5.2 |
| CD19+B Cell Percent  (%) | 4-5 y.o. 13-31  6-7 y.o. 11-28  8-9 y.o. 13-27 | 23 | 22 | 19 | 16.8 | 19.1 | 17.6 |
| CD19+B Cell Number (10^3/µL) | 4-5 y.o. 421-1,397  6-7 y.o. 249-865  8-9 y.o. 270-860 | 662 | 612 | 448 | 281 | 376 | 332 |
| CD3-CD56CD 16+Percent (%) | 4-5 y.o. 3-19  6-7 y.o. 5-21  8-9 y.o. 4-17 | 8.0 | 7.0 | 7.0 | 10.1 | 8.1 | 11.0 |
| CD3-CD56CD 16+Number (10^3/µL) | 4-5 y.o. 123-785  6-7 y.o. 128-474  8-9 y.o. 100-480 | 230 | 195 | 165 | 169 | 159 | 208 |
| CD4 to CD8 Ratio | 4-5 y.o. 0.7-2.9  6-7 y.o. 0.7-2.6  8-9 y.o. 0.7-2.6 | 1.37 | 1.76 | 1.85 | 1.97 | 1.82 | 1.52 |
| CD3+CD56CD16+Percent (%) | 4-5 y.o. 0-10  6-7 y.o. 0-10  8-9 y.o. 0.5-23 | 1.0 | 1.0 | 1.0 | 1.8 | 2.3 | 2.6 |
| CD3+CD56CD16+Number (10^3/µL) | 4-5 y.o. 0-200  6-7 y.o. 0-200  8-9 y.o. 0-766 | 29 | 28 | 24 | 30 | 45 | 49 |
| CD19+CD27-IgM+IgD+Percent (%) | 4-5 y.o. 31-91  6-7 y.o. 43-89  8-9 y.o. 43-89 | 81.1 | TNP | TNP | 91.5 | 87.3 | 87.1 |
| CD19+CD27-IgM+IGD+Number (10^3/μL) | 4-5 y.o. 12-336  6-7 y.o. 3-383  8-9 y.o. 3-383 | 464 | TNP | TNP | 243 | 333 | 337 |
| CD19+CD27+Percent  (%) | 4-5 y.o. 0-58  6-7 y.o. 2-36  8-9 y.o. 2-36 | 7.9 | TNP | TNP | 5.8 | 5.7 | 11.3 |
| CD19+CD27+Number (10^3/μL) | 4-5 y.o. 0-204  6-7 y.o. 2-122  8-9 y.o. 2-122 | 45 | TNP | TNP | 15 | 22 | 44 |
| CD19+CD27+IgM+IgD+  Percent (%) | 4-5 y.o. 0-40  6-7 y.o. 0-19  8-9 y.o. 0-19 | 5.5 | TNP | TNP | 5.0 | 5.4 | 10.8 |
| CD19+CD27+IgM+IgD+  Number (10^3/μL) | 4-5 y.o. 0-140  6-7 y.o. 0-50  8-9 y.o. 0-50 | 31 | TNP | TNP | 13 | 21 | 42 |
| CD19+CD27+IgM+IgD-Percent (%) | 4-5 y.o. 0-4  6-7 y.o. 0-5  8-9 y.o. 0-5 | 0.1 | TNP | TNP | 0 | 0 | 0.1 |
| CD19+CD27+IgM+IgD-Number (10^3/μL) | 4-5 y.o. 0-10  6-7 y.o. 0-8  8-9 y.o. 0-8 | 1.0 | TNP | TNP | 0 | 0 | 0 |
| CD19+CD27+IgM-IgD-Percent (%) | 4-5 y.o. 0-30  6-7 y.o. 0-22  8-9 y.o. 0-22 | 2.3 | TNP | TNP | 0.7 | 0.2 | 0.3 |
| CD19+CD27+IgM-IgD-Number (10^3/μL) | 4-5 y.o. 0-94  6-7 y.o. 0-74  8-9 y.o. 0-74 | 13 | TNP | TNP | 2.0 | 1.0 | 1.0 |
| CD19+CD20+Percent  (%) | 4-5 y.o. 97-100  6-7 y.o. 96-100  8-9 y.o. 96-100 | 99.3 | TNP | TNP | 99.8 | 98.5 | 99.9 |
| CD19+CD20+Number (10^3/μL) | 4-5 y.o. 42-524  6-7 y.o. 22-546  8-9 y.o. 22-546 | 568 | TNP | TNP | 265 | 376 | 386 |
| CD19+CD38+Bright IgM+Bright Percent (%) | 4-5 y.o. 0-7  6-7 y.o. 0-6  8-9 y.o. 0-6 | 5.9 | TNP | TNP | 14.3 | 7.5 | 9.1 |
| CD19+CD38+Bright IgM+Bright Number (10^3/μL) | 4-5 y.o. 0-21  6-7 y.o. 0-18  8-9 y.o. 0-18 | 34 | TNP | TNP | 38 | 29 | 35 |
| CD19+CD38+Bright IgM- Percent (%) | 4-5 y.o. 0-3  6-7 y.o. 0-3  8-9 y.o. 0-3 | 0.9 | TNP | TNP | 1.1 | 0.5 | 0.1 |
| CD19+CD38+Bright IgM- Number (10^3/μL) | 4-5 y.o. 0-6  6-7 y.o. 0-7  8-9 y.o. 0-7 | 5.0 | TNP | TNP | 3.0 | 2.0 | 0 |
| CD19+CD38+Bright Percent (%) | 4-5 y.o. 0-16  6-7 y.o. 0-14  8-9 y.o. 0-14 | 15.4 | TNP | TNP | 21.7 | 17.1 | 16.5 |
| CD19+CD38+Bright Number (10^3/μL) | 4-5 y.o. 0-50  6-7 y.o. 1-39  8-9 y.o. 1-39 | 88 | TNP | TNP | 58 | 65 | 64 |
| CD19+CD21+ Percent  (%) | 4-5 y.o. 88-100  6-7 y.o. 86-100  8-9 y.o. 86-100 | 94.2 | TNP | TNP | 91.9 | 86.5 | 95.9 |
| CD19+CD21+ Number (10^3/μL) | 4-5 y.o. 35-508  6-7 y.o. 17-522  8-9 y.o. 17-522 | 539 | TNP | TNP | 244 | 330 | 371 |
| CD19+CD38+CD21+ Percent (%) | 4-5 y.o. 49-100  6-7 y.o. 57-96  8-9 y.o. 57-96 | 90.2 | TNP | TNP | 88 | 87.5 | 87.2 |
| CD19+CD38+CD21+ Number (10^3/μL) | 4-5 y.o. 16-431  6-7 y.o. 16-425  8-9 y.o. 16-425 | 516 | TNP | TNP | 234 | 334 | 337 |
| CD19+CD38-/LowCD21-/Low Percent (%) | 4-5 y.o. 0-8  6-7 y.o. 0-7  8-9 y.o. 0-7 | 1.6 | TNP | TNP | 1.7 | 1.7 | 1.7 |
| CD19+CD38-/LowCD21-/Low Number (10^3/μL) | 4-5 y.o. 0-19  6-7 y.o. 0-21  8-9 y.o. 0-21 | 9.0 | TNP | TNP | 5.0 | 6.0 | 7.0 |
| Proliferation to Mitogens & Antigens | NA | Normal | TNP | Normal | Normal | Normal | TNP |

*IgG obtained while patient’s daughter was receiving IgG replacement therapy

+ANC obtained while patient’s daughter was receiving G-CSF therapy

TNP: test not performed
